# Dietary Patterns and Their Association with Anxiety Symptoms among Older Adults: The ATTICA Study

**DOI:** 10.3390/nu11061250

**Published:** 2019-05-31

**Authors:** Maria F. Masana, Stefanos Tyrovolas, Natasa Kollia, Christina Chrysohoou, John Skoumas, Josep Maria Haro, Dimitrios Tousoulis, Charalambos Papageorgiou, Christos Pitsavos, Demosthenes B. Panagiotakos

**Affiliations:** 1 Parc Sanitari Sant Joan de Déu, Universitat de Barcelona, Fundació Sant Joan de Déu, 08007 Barcelona, Spain; mfmasana@gmail.com (M.F.M.); jmharo@pssjd.org (J.M.H.); 2 Instituto de Salud Carlos III, Centro de Investigación Biomédica en Red de Salud Mental, CIBERSAM, Monforte de Lemos 3-5, Pabellón 11, 28029 Madrid, Spain; 3 Facultat de Medicina, Universitat de Barcelona, Casanova, 143, 08036 Barcelona, Spain; 4 Department of Science of Dietetics and Nutrition, School of Health Science and Education, Harokopio University, 17671 Athens, Greece; natkollia@yahoo.gr (N.K.); dbpanag@hua.gr (D.B.P.); 5 First Cardiology Clinic, School of Medicine, University of Athens, 15772 Athens, Greece; chrysohoou@usa.net (C.C.); skoumasj@gmail.com (J.S.); tousouli@med.uoa.gr (D.T.); cpitsavo@med.uoa.gr (C.P.); 6 First Psychiatry Clinic, School of Medicine, University of Athens, 15772 Athens, Greece; cpapage@eginitio.uoa.gr

**Keywords:** snack, sugars, carbohydrate, energy intake, anxiety, older adults, Attica study, Greece

## Abstract

By 2050, the global population aged 60 years and over is expected to reach nearly 2.1 billion and affective disorders might be also expected to increase. Although nutrition has been related with affective disorders, there is a lack of studies assessing the relation between dietary habits and anxiety among European and Mediterranean older populations. In the present study, we aimed to evaluate the association between dietary habits, energy intake, and anxiety symptoms using data from 1128 Greek older adults (>50 years) without pre-existing cardiovascular disease (CVD) or any other chronic disease who participated in the ATTICA study. Various socio demographic lifestyle, bio-clinical (e.g., blood pressure), and psychological (e.g., depression) characteristics were used, and dietary habits as well as energy intake were calculated using standard procedures. Older people with anxiety were more likely to be sedentary, to be smokers, and to show symptoms of depression. The saturated fat and added sugars (SFAS) dietary pattern was associated with higher anxiety levels (non-standardized b (95% CI): 5.82 (0.03 to 11.61)). No association between energy intake tertiles and anxiety levels pictured in the later regression model. Moreover, female gender, family status, and depression were positively related to anxiety. Therefore, promoting healthy dietary habits could reduce anxiety symptoms of the older adults.

## 1. Introduction

The global population aged 60 years or over is expected to reach nearly 2.1 billion by 2050 due to reductions in fertility and a rise in life expectancy [1] resulting in an increased risk for non-communicable diseases such as cancer, dementia, and affective disorders (anxiety and depression). Particularly, affective disorders might be expected to be increased because older people living alone is continuously rising as a consequence of changes in societies, such as falling family size, family structure and less multigenerational living arrangements [2].

The estimated global prevalence for anxiety disorders was 7.3% (95% CI 4.8–10.9%) and considering socioeconomic factors and culture, the prevalence ranged from 5.3% (3.5–8.1%) to 10.4% (7.0–15.5%) in African and Euro/Anglo cultures, respectively [3]. However, anxiety disorders are frequently under-recognized and misdiagnosed. Consequently, individuals with an undiagnosed or misdiagnosed anxiety disorder may not receive the appropriate treatment, suffering a deterioration of quality of life and social ability [4]. Several risk factors have been related to anxiety, but the emergence of a health and social crisis due to the financial crisis and strict fiscal austerity in Europe (e.g., Greece, Portugal, and Spain) could have contributed to worsen mental health [5]. For instance, in Greece, worse health status was reported in older adults due to unhealthy changes in their lifestyle behaviours [6] and anxiety disorders were also reported to be the fourth cause of years lost due to disability (YLDs) [7].

Depression has shown to be the most common comorbid disorder with anxiety disorders [8], but a bidirectional prospective relationship with one another was also reported [9]. Although the relationship between anxiety and cognition remains unclear, some studies have shown that anxiety was associated with cognitive decline, specifically on spatial and verbal working memory [10], influencing positively in the progression of Mild Cognitive Impairment (MCI) to dementia [11,12]. Some other chronic diseases, such as gastrointestinal disorders (e.g., irritable bowel syndrome) [13], diabetes [14] and thyroid disorders [15] have been also related to anxiety disorders or/and elevated anxiety symptoms. Moreover, patients with cardiovascular disease (CVD) are more likely to show prevalent anxiety disorders compared to the general population, but anxiety disorders have been also reported to increase the CVD risk [16]. For instance, anxiety levels were directly associated with the 10 y CVD incidence (OR (95%CI): 1.03 (1.0–1.1)) in older Greek adults without previous CVD history [17]. Some common pathophysiological mechanisms seem to share anxiety and the interrelated comorbidities, such as increase of corticotropin-releasing factor (CRF) levels, dysregulation of the hypothalamic-pituitary-adrenal (HPA) axis [18], activation of the sympathetic nervous system, and increase of levels of proinflammatory cytokines [19,20].

In recent years, an increasing number of studies are providing evidence for diet as a modifiable risk factor for mental health problems, such as depression and anxiety, however there is a lack of data regarding the older adults, a vulnerable population subgroup that appears to have high prevalence of mental disorders. For instance, a dietary pattern characterized by consumption of vegetables, fruit, beef, lamb, fish, and whole-grain foods has been related to a lower risk of a diagnosis of anxiety, while a western dietary pattern characterized by consumption of foods such as meats and sugar has been associated with more psychiatric symptoms [21]. Later studies also found that an inadequate and poor diet quality (i.e., snack patterns, animal foods) [22] and high intake of alcohol [23] can contribute to an inadequate intake of nutrients leading to mental health problems. 

To date, there is a lack of studies assessing the association between dietary habits, energy intake, and anxiety symptoms among older adults and specifically, among European and Mediterranean older populations. Therefore, the aim of the current study was to evaluate the relation between dietary patterns, energy intake, and anxiety in a sample of older people (50+ years old) living in the Attica region.

## 2. Methods

### 2.1. Study Population

In brief, ATTICA is a prospective, population-based, cohort study performed in Attica (Athens metropolitan region, Greece), which recruited 3042 non-institutionalized adults (Caucasians; women/men: 1528/1514; age: ≥18 years) without previous CVD. The enrolment of the participants was carried out during 2001–2002, and after that, two follow-up waves followed 5 (in 2006) and 10 years (in 2012) later. Random, multistage sampling based on the age and gender distribution of the reference population, as defined by the Hellenic National Statistical Service Census Survey of 2001, was applied. Sampling procedures anticipated enrolling only one participant per household, while institutionalized individuals were excluded from study participation. All participants underwent detailed baseline assessments which included medical history, physical examination, and blood sampling for biochemical measurements. Baseline CVD was excluded in all participants by the study physicians [24]. For the purposes of the present work, only baseline data for the study’s sample of over 50 years old (i.e., *n* = 1128) men and women were used (there was no psychological assessment at follow-up examinations).

### 2.2. Sociodemographic and Life-Style Variables

The study questionnaire included demographic information such as age, gender, family status (married, divorced, widowed), financial status, and education level. The level of education (as a proxy of social status) was determined by years of schooling and classified into 3 groups: (1) <9 years; (2) up to high school or technical college (10–14 years); (3) university. Mean annual income during the past 3 years was also recorded.

Current smokers were defined as those who smoked at least one cigarette/day, never smokers as those who had never tried a cigarette in their life, and former smokers as those who had stopped smoking for at least one year. Occasional smokers (7 cigarettes/week) were recorded and combined with current smokers because of their small sample size. In order to evaluate more accurately the smoking habits, we calculated the pack-years (cigarette packs/day × years of smoking), adjusted for a nicotine content of 0.8 mg/cigarette.

The physical activity level of each participant was also assessed at baseline using the International Physical Activity Questionnaire (IPAQ; participants reporting no physical activities or exercise on the IPAQ were classified as physically inactive) [25]. 

### 2.3. Clinical and Biochemical Assessments

Standardized measurements of anthropometric parameters were performed by trained study researchers, including body weight and height, BMI (kg/m^2^), and WC (cm). Resting arterial blood pressure (BP, average of 3 recordings in sitting position) was also measured, and participants exhibiting average BP ≥ 140/90 mmHg or taking antihypertensive medication(s) were categorized as hypertensive.

At baseline, fasting blood samples were obtained from all participants after overnight fasting. Triglyceride (TG), total cholesterol (TC) and high-density lipoprotein-cholesterol (HDL-C) levels were measured by a chromatographic enzymatic method using a Technicon automatic analyzer RA-1000 (Dade Behring, Marburg, Germany; corresponding intra- and inter-assay coefficients of variation (CV) were <4%, <9%, and <4%, respectively). Low-density lipoprotein-cholesterol (LDL-C) was calculated by the Friedewald formula [26]. Hypercholesterolemia was defined as TC > 200 mg/dL or treatment with lipid-lowering drug(s). Moreover, fasting blood glucose (FBG) levels were measured by a Beckman glucose analyzer (Beckman Instruments, Fullerton, CA, USA) and subjects with FBG >125 mg/dL or on antidiabetic treatment were classified as having diabetes.

### 2.4. Dietary Assessment

All participants underwent a detailed baseline dietary evaluation through the EPIC-Greek questionnaire [27], which is a validated semi-quantitative food-frequency questionnaire that was kindly provided by the Unit of Nutrition of Athens Medical School. The energy intake in kcal/day was also calculated based on the participants’ responses in this questionnaire. Energy intake tertiles were also created, those in the 1st energy tertile were the participants consuming <1840 kcals/day.

All participants were asked to report the average intake (per week or day) of several food items that they consumed (during the last 12 months). Then, the frequency of consumption was quantified approximately in terms of the number of times a month this food was consumed. Thus, daily consumption multiplied by 30 and weekly consumption multiplied by 4 and a value of 0 was assigned to food items rarely or never consumed. Alcohol consumption was measured in wineglasses (100 ml) and quantified by ethanol intake (grams per drink). One wineglass was considered equal to 12% ethanol concentration. 

A dietary pyramid was developed to describe the Mediterranean dietary pattern [28]. This dietary pattern consist of: (a) daily consumption of non-refined cereals and products (whole grain bread, pasta, brown rice, etc.), vegetables (2–3 servings/day), fruits (6 servings/day), olive oil (as the main added lipid) and dairy products (1–2 servings/day); (b) weekly consumption of fish (4–5 servings/week), poultry (3–4 servings/week), olives, pulses, and nuts (3 servings/week), potatoes, eggs and sweets (3–4 servings/week) and monthly consumption of red meat and meat products (4–5 servings/month). The Mediterranean diet (MedDiet) is also characterized by moderate consumption of wine (1–2 wineglasses/day) and high monounsaturated to saturated fat ratio (>2). 

A special diet score that assessed adherence to MedDiet was calculated. In particular, we assigned a score of 0 for rare or no consumption of food items that are close to this dietary pattern, 1 for 1 to 4 times/month, 2 for 5 to 8 times/month, 3 for 9 to 12 times/month, 4 for 13 to 18 times/month and 5 for almost daily consumption. On the other hand, for the consumption of foods that are away from this traditional diet, like meat and meat products, we assigned the opposite scores (i.e. 0 for almost daily consumption to 5 for rare or no consumption). For alcohol, we assigned a score of 5 for consumption of less than 3 wineglasses per day, a score of 0 for consumption of more than 7 wineglasses/day and scores of 1 to 4 for consumption of 3, 4–5, 6 and 7 wine glasses per day. 

The calculation of the dietary inflammatory load of the participants’ diet was according to the methodology and the rationale of the Dietary Inflammation Index (DII) that has been previously proposed by Shivappa and colleagues [29]. Thus, a Dietary Anti-Inflammation Index (D-AII) was developed based on participants' dietary habits. Detailed information on the D-AII has been reported in a previous ATTICA publication, by Georgousopoulou et al [30].

Aside from the classic indexes of MedDiet and D-AII, additional dietary patterns were defined for the daily dietary intake of the participants. Three main components were extracted using the principal components (PC) methods (Table 1). These were: (A) The lacto-fish-vegetarian (LFV) dietary pattern including the food components of vegetables, cereals, fruits, legumes, fish, and dairy products; (B) The meat-eaters dietary pattern including food components of all kinds of meat, read meat, and poultry; (C) The saturated fat and added sugars (SFAS) dietary pattern including the food components of sweets, soft drinks, nuts, and potatoes.

### 2.5. Psychological Assessments

Anxiety levels were assessed using the validated Greek translation of the 20 item, self-report State-Trait Anxiety Inventory (STAI) [31], the total score of which ranges from 20 to 80. Higher scores in this scale are indicative of more severe anxiety, and according to Spielberger’s criteria, a score of 40 or higher reflects clinically relevant symptoms of anxiety. 

Depressive symptomatology was assessed using a translated and validated version of the Zung Self-Rating Depression Scale (ZDRS) [32]. The ZDRS consists of 20 items that cover affective, psychological, and somatic symptoms, and ranges from 20 to 80. A subject with a ZDRS score below 50 is considered normal but with a score of 50 to 59, 60 to 69, or 70 or above is considered to suffer from mild, moderate, or severe depression, respectively. 

Cognitive distortion symptomatology was assessed using a validated version of the cognitive distortions scale (CDS) [33]. This tool captures cognitive symptoms or distortions among individuals who have experienced inter-personal victimization: self-criticism, self-blame, helplessness, hopelessness, and preoccupation with danger.

### 2.6. Bioethics

The ATTICA study was approved by our institutional ethics committee and conformed to the ethical guidelines of the 1975 declaration of Helsinki. All participants were informed about the study protocol, and they provided written signed consent.

### 2.7. Statistical Analysis

To define the dietary patterns, factor analysis using the PC was applied [34]. Principal components were retained if their eigenvalues were greater than 1.0, a threshold that is commonly used as a cut-off to identify meaningful patterns. The resulting components were interpreted based on the variables with loadings above 0.3. Moreover, the Kaiser-Meyer-Olkin criterion was used as a measure of variables (components) inter-correlation and used as an indicator of internal consistency.

Normally distributed continuous variables are presented as mean values ± SD, and categorical variables as frequencies. Normality was tested using the Shapiro-Wilk criterion; the non-normally distributed variables are presented as median and 1st, 3rd tertile. Associations between categorical variables were tested by the chi-square test, whereas between continuous variables by the Pearson r or Spearman’s rho coefficients for normally distributed and skewed variables, respectively. Continuous variables were tested for normality via P-P plots. For normally distributed variables, comparisons were performed by the student’s t-test, after controlling for equality of variances by the Levene’s test. For continuous variables without normal distribution, comparisons were performed by the non-parametric Mann-Whitney U-test. Previous literature was used as a guide for the selection of variables used for adjustment between dietary patterns and anxiety [35,36,37]. Multiple linear regression analyses were performed in order to evaluate the association between anxiety as the dependent outcome and a participant´s adherence to dietary patterns, adjusted for multiple confounders. All *p*-values are based on two-sided tests. Statistical analyses were performed with the Statistical Package for Social Sciences version 22 (SPSS Inc., Chicago, IL, USA).

## 3. Results

In Table 1 the component loadings and the eigenvalues from the factor analysis are presented. The size of eigenvalues strongly suggests the formation of three distinct dietary patterns, explaining 51.78% of the total variance of the information. The first dietary pattern could be characterized as the “lacto-fish-vegetarian”, the second as the “meat-eaters” and the third as the “saturated fat and added sugars”. 

The sociodemographic, lifestyle, and clinical characteristics of participants are presented by anxiety status in Table 2. Compared to the participants without anxiety, those with anxiety were more likely to be sedentary (*p* = 0.001), smokers (*p* = 0.027), and suffered depressive symptoms (*p* < 0.001). The rest of measured variables (i.e., age, gender, education, income, family status, diet, energy intake, BMI, hypertension, diabetes, hypercholesterolemia, and cognitive distortion) were not significantly associated with the level of anxiety. 

As a first step, results of multi-adjusted analysis assessing the energy intake by tertiles on anxiety levels are presented in Table 3, without adjusting for dietary patterns. Initially the association between energy intake (kcals/day) as a continuous variable and anxiety levels was tested. Based on this analysis, it was observed that the higher levels of energy intake were positively related with higher anxiety levels (non-standardized b (95% CI): 0.01 (0.003 to 0.2)), after various adjustments (i.e., sex, smoking habits, physical activity, etc.) (data shown only in text). When the analysis was applied by energy intake tertiles, it was shown that the 1st energy intake tertile had an independent inverse association with anxiety levels as compared with the highest one (3rd tertile) (non-standardized b (95% CI): −11.65 (−22.83 to −0.48), *p* = 0.04). The 2nd energy intake tertile as compared with the 3rd tertile, was no related with anxiety levels (non-standardized b (95% CI): −7.61 (−18.55 to 3.34), *p* = 0.16). In addition, female gender (non-standardized b (95% CI): 11.96 (0.53 to 23.38), *p* = 0.04), family status (non-standardized b (95% CI): 18.17 (4.56 to 31.77), *p* = 0.012), and depression (non-standardized b (95% CI): 1.07 (0.27 to 1.87), *p* = 0.01) were positively related to higher anxiety levels. Cognitive distortion (non-standardized b (95% CI): 0.20 (0.09 to 0.32), *p* = 0.001) was also observed to be associated with the presence of anxiety symptoms, while physical activity was inversely related with anxiety levels (non-standardized b (95% CI): −8.58 (−15.90 to −1.25), *p* = 0.02). Finally, the results remained similar when basic metabolic rate was inserted as a confounding variable in the model.

As a next step, Table 4 illustrates the results from multiple linear regression analysis that evaluated the association between dietary patterns, energy intake and anxiety (models I and II). After adjusting for sociodemographic, lifestyle and clinical characteristics, as in Table 3, plus for confounding due to obesity, central obesity (waist circumference), and energy intake, the dietary pattern characterized by the consumption of saturated fats and added sugars (SFAS dietary pattern) was consistently associated with higher anxiety levels (non-standardized b (95% CI): 5.82 (0.03 to 11.61), *p* = 0.04) (Model II). Moreover, anxiety was positively associated with female gender (non-standardized b (95% CI): 21.06 (3.19 to 38.94), *p* = 0.02), and family status (non-standardized b (95% CI): 19.83 (2.47 to 37.19), *p* = 0.02). In addition, depressive symptomatology was related with higher level of anxiety (non-standardized b (95% CI): 1.88 (0.48 to 3.28), *p* = 0.01). Finally, LFV dietary pattern as well as meat-eaters and SFAS dietary patterns were replaced with the D-AII, a dietary index that is picturing pro- and anti- inflammatory dietary habits. In this additional analysis, there was not association between the D-AII and the anxiety levels (*p* = 0.94) (data shown only in text).

## 4. Discussion

While anxiety is multi-factorial and mostly related with psychological, social and biological aspects [38], little is known about the contribution of holistic dietary patterns as preventive means of anxiety in older populations. Through factor analysis, three distinct dietary patterns were pictured (the LFV, the meat-eaters and the SFAS dietary patterns) among the older adults (50+ years old) of the ATTICA study. The present study reported that at an initial step, energy intake was related with anxiety levels among older adults. However, when the abovementioned dietary patterns were inserted in the analysis, it was reported that the SFAS dietary pattern was associated with higher levels of anxiety among older adults, while energy intake was no longer significant. Notably, this relation was independent of well-established risk factors such as depression, physical activity, and cognitive distortion. To the best of our knowledge, this is among the first studies to evaluate the association between dietary patterns, energy intake, and anxiety levels using a non-CVD, European, older population. These results point to the importance of specific dietary patterns in relation with anxiety and the public health actions needed to be taken with western older populations in which the burden of cognitive and mental disorders is increasing at alarming rates.

Older participants with anxiety from the Attica region in Greece were more likely to be smokers, sedentary and presented depressive symptoms, and this results are in agreement with the results of studies found in the literature. In particular, systematic reviews have reported that smoking [39] and depression [9] might be risk factors for the development of anxiety, but a bidirectional relationship has also been observed. Moreover, previous studies have suggested that a sedentary behavior (i.e., sitting time and screen time) is related to an increased risk for anxiety [40], and higher levels of anxiety were associated with increased diabetes risk among women but not among men [41].

Multi-adjusting analysis revealed an association between socio-economic and lifestyle factors and mental health problems with anxiety levels in older adults. In previous studies, associations between socio-economic and lifestyle factors have also reported. For instance, women with anxiety have reported lower socioeconomic status and income compared to men [42], and other risk factors such as stressful life events, chronic diseases, physical inactivity, depression, insomnia, and lower cognitive function have also been related to anxiety [43]. Regular physical activity has been related to reduced anxiety due to its impact on some pathological mechanisms, such as modulation of HPA and an increase of brain-derived neurotrophic factor (BDNF) levels and β-endorphins [44]. Smoking have been reported to activate the HPA axis increasing adrenocorticotropic hormone (ACTH) and cortisol [45], which have been related with anxiety [46]. However, in our fully adjusted regression analysis, physical activity, financial status and smoking habits did not remain significant. Anxiety has been also related to worse performance in some cognitive domains, such as spatial and verbal working memory [10], and bidirectional associations between anxiety symptomology and processing speed and attention have also reported in older adults [47]. Literature suggests that anxiety is highly comorbid with depression due to the overlap of symptomatology of both disorders increasing its severity and chronicity, and consequently, a worsening in the quality of life [48], but an inverse relationship between anxiety and reduced cognitive function have been also observed and explained by comorbid depression in non-demented elderly general population [49]. Therefore, older adults with healthy habits such as regular physical activity, healthy sleeping routine, and no smoking could contribute to maintain a good mental health reducing affective disorders, and consequently, reducing the deterioration of cognitive function. 

Dietary habits are usually estimated using PC analysis and are reflecting the real behavior of the population [50]. To date various dietary patterns have been identified among older populations. Healthy dietary patterns (i.e., higher consumption of vegetables, fruits, poultry and fish), have been related with better quality of life, self-reported health, lower burden of morbidity as well as higher survival among the older adults [51,52]. However, until today, research on the effect of dietary patterns on the anxiety levels among older adults is sparse. In our analysis we extracted three major dietary patters (the LFV, the meat-eaters and the SFAS dietary patterns). Of them, only the SFAS dietary pattern characterized by the consumption of added sugars and saturated fats was related with higher anxiety levels, while no association was reported between the LFV and the meat-eaters dietary patterns. Furthermore, no relation was reported between anti-inflammatory dietary patterns (D-AII) and anxiety. These associations are partly in line with the literature, where mixed results are reported. For example, a previous ATTICA work focused only in women reported that increased consumption of sweets and meat products were associated with higher anxiety levels [35]. Other recent studies report that diets high in sugars, processed foods (i.e., meat products), and/or fats are related with higher anxiety levels through alterations of glucose, protein and energy homeostasis, and increases in inflammatory cytokines (i.e., IL-6, IL-1β, TNFα) and corticosterone [53,54]. Inverse associations have also been reported between anti-inflammatory dietary patterns [36], high intake of fruit and vegetables [37], and anxiety. Our analysis between energy intake tertiles and anxiety referred no association in the final regression model, although, some animal studies report an anxiolytic caloric restriction (CR) effect [55] contributing beneficially to brain microstructure integrity [56]. Further studies, especially with longitudinal data, are needed on dietary habits and anxiety levels among older adults to confirm our findings.

Anxiety disorders in high-income countries for the population over 70 years old in 2017 account for 1.41% of total YLDs (https://vizhub.healthdata.org/gbd-compare/). These associations combined with the fact that Western societies are ageing along with the increased epidemic of depression and cognitive problems raise major concerns about the need for early non-pharmacological measures (and promotion of lifestyle changes) in order to prevent anxiety among the older populations. 

### Strengths and limitations

The present study has several strengths since it is one of the few studies to evaluate the relation between dietary habits, energy intake, and anxiety in Mediterranean older adults. However, some limitations deserve mentioning. It should be acknowledged that the baseline/entry study examination was conducted once and hence, may be susceptible to a certain degree of measurement error. This observational study may have potential recall bias due to its cross-sectional design, and consequently, the nature of the study limits the possibility for etiological conclusions between dietary habits and anxiety. The assessment of dietary habits using as a tool the FFQ is known for certain bias. Furthermore, there might be several misinterpretations since the components extracted from a PC are sometimes subjective.

## 5. Conclusions

Our results report that a dietary pattern characterized by saturated fats and added sugars (SFAS) seems to be related to higher anxiety in older adults living in Greece while energy intake was not related with anxiety in the fully adjusted model. Further studies and longitudinal data analysis are needed to better address this interesting question on the role of dietary habits in the symptomatology of anxiety among older populations.

## Figures and Tables

**Table 1:** Factor loadings of the food components included in the extracted dietary patterns.

Food Components	LFV Dietary Pattern	Meat-Eaters Dietary Pattern	SFAS Dietary Pattern
Vegetables	0.756	–	–
Fruit	0.653	–	–
Cereals	0.622	–	–
Legumes	0.609	–	–
Fish	0.579	–	–
Dairy Products	0.435	–	–
Total Meat	–	0.941	–
Red Meat	–	0.828	–
Poultry	–	0.708	–
Sweets	–	–	0.675
Soft Drinks	–	–	0.649
Nuts	–	–	0.648
Potatoes	–	–	0.523
Cumulative Variance explained (%)	25.675	41.609	51.788

Abbreviations: LFV, lacto-fish-vegetarian; and, SFAS, saturated fat and added sugars. In each factor only the components with values > 0.30 are included in the table.

**Table 2:** Baseline characteristics of the study sample 50+ years old, by anxiety status.

Characteristic	Total	No Anxiety	Anxiety	*p*
Sample size (*n*)	758	149	609	
Age (Mean ± SD)	59.67 ± 8.28	54.11 ± 4.31	54.68 ± 3.39	0.372
Sex				0.286
Female	41.6	37.3	45.9	
Male	58.4	62.7	54.1	
Education status (years of school) (Mean ± SD)	10.53 ± 3.96	12.43 ± 3.65	11.36 ± 4.15	0.100
Financial status (%)				0.151
Bad	12.8	8.0	17.6	
Poor	17.4	18.7	16.2	
Good	47.7	45.3	50.0	
Very good	22.1	28.0	16.2	
Family status (%)				0.580
Never married	10.7	10.7	10.08	
Married	78.5	80.0	77.0	
Divorced	7.4	8.0	6.8	
Widowed	3.4	1.3	5.4	
Current smoking (%)	46.3	37.3	55.4	0.027
Physically active (%)				0.001
Sedentary	52.3	38.7	66.2	
Physically active	47.7	61.3	33.8	
MedDiet (0–55)	23.02 ± 6.17	24.27 ± 5.25	24.03 ± 6.36	0.808
SFAS dietary pattern (Mean ± SD)	−0.32 ± 0.86	−0.189 ± 0.756	−0.33 ± 0.81	0.337
Meat-eaters dietary pattern (Mean ± SD)	−0.24 ± 0.76	−0.35 ± 0.55	−0.22 ± 0.77	0.294
LFV dietary pattern (Mean ± SD)	0.25 ± 0.98	0.29 ± 0.89	0.146 ± 1.09	0.434
Energy intake (kcal/day)	2409.38 ± 1019.19	2120.10 ± 693.15	2048.51 ± 855.46	0.617
D-AII (10–77)	32.61 ± 6.34	32.81 ± 6.04	32.44 ± 6.62	0.72
BMI (kg/m^2^)	27.60 ± 4.25	26.87 ± 4.11	26.48 ± 3.29	0.532
Waist circumference (cm)	94.64 ± 18.87	95.58 ± 21.12	93.70 ± 16.70	0.57
Hypertension (%)	45.9	43.1	48.6	0.498
Diabetes (%)	10.1	8.0	12.2	0.399
Hypercholesterolemia (%)	51.0	46.7	55.4	0.286
Zung score (Mean ± SD)	35.17 ± 7.57	31.65 ± 6.30	38.54 ± 6.88	<0.001
Cognitive distortion score (Mean ± SD)	17.84 ± 24.35	11.36 ± 15.11	24.48 ± 30.35	0.073

Abbreviations: MedDiet, mediterranean diet; BMI, body mass index; LFV, lacto-fish-vegetarian; SFAS, saturated fat and added Sugars; diet anti-inflammatory index (D-AII).

**Table 3:** Correlates of anxiety among adults aged ≥ 50 years estimated by multivariable linear regression, in the ATTICA study, *n* = 758.

Variable	Non-Standardized b	95% CI
Female vs. male sex	11.96 *	0.53, 23.38
Education status (per 1 year)	0.87	−0.10, 1.85
Family status (married vs. other status)	18.17 *	4.56, 31.77
Financial status (high vs. low-medium)	−9.66 *	−19.32, −0.002
Physical activity (active vs. sedentary)	−8.58 *	−15.90, −1.25
Current smoking (yes vs. no)	−1.50	−9.40, 6.41
Energy intake (kcals)		
1st vs. 3rd tertile	−11.65 *	−22.83, −0.48
2nd vs. 3rd tertile	−7.61	−18.55, 3.34
BMI (per 1 kg/m^2^)	0.91	−0.24, 2.06
Hypertension (yes vs. no)	1.68	−5.31, 8.68
Diabetes (yes vs. no)	1.88	−22.32, 26.09
Hypercholesterolemia (yes vs. no)	1.95	−5.16, 9.06
Depression (Zung scale)	1.07 *	0.27, 1.87
Cognitive distortion scale	0.20 **	0.09, 0.32

Data are presented as non-standardized coefficients (b) and their 95% confidence intervals (CIs). * *p* < 0.05, ** *p* < 0.01. Abbreviations: BMI, body mass index. Model is adjusted for all the covariates in the table.

**Table 4:** Correlates of anxiety among adults aged ≥ 50 years estimated by additive multivariable linear regression (Model I and Model II), in the ATTICA study, *n* = 758.

Variable	Model I		Model II	
	Non-Standardized b	95% CI	Non-Standardized b	95% CI
Female vs. male sex	21.77	7.25, 36.28	21.06	3.19, 38.94
Education status (per 1 year)	1.92	0.29, 3.56	1.78	−0.07, 3.65
Family status (married vs. other status)	20.72	4.81, 36.63	19.83	2.47, 37.19
Financial status (high vs. low-medium)	−9.33	−19.61, 0.95	−9.84	−21.22, 1.53
Physical activity (active vs. sedentary)	−4.32	−13.35, 4.69	−4.00	−14.42, 6.41
Current smoking (yes vs. no)	−3.54	−12.30, 5.21	−2.42	−12.50, 7.66
LFV dietary pattern	−1.96	−9.05, 5.12	−1.20	−9.29, 6.88
Meat-eaters dietary pattern	3.41	−3.80, 10.62	3.33	−4.92, 11.59
SFAS dietary pattern	5.62	1.21, 10.02	5.82	0.03, 11,61
Obesity (yes vs. no)	19.22	0.51, 37.94	18.00	−4.52, 40.54
Waist circumference (cm)	−0.17	−0.45, 0.12	−0.16	−0.51, 0.18
Hypertension (yes vs. no)	5.70	−2.47, 13.87	5.17	−3.73, 14.08
Diabetes (yes vs. no)	−1.36	−26.13, 23.39	1.71	−26.53, 29.96
Hypercholesterolemia (yes vs. no)	1.96	−5.60, 9.54	2.67	−6.04, 11.40
Depression (Zung scale)	1.97	0.76, 3.17	1.88	0.48, 3.28
Cognitive distortion scale	0.14	−0.04, 0.37	0.16	−0.07, 0.39
Energy intake (kcals)	–	–		
1st vs. 3rd tertile	–	–	−0.48	−18.98, 18.02
2nd vs. 3rd tertile	–	–	−2.97	−16.80, 10.86

Data are presented as non-standardized coefficients (b) and their 95% confidence intervals (CIs). Abbreviations: LFV, lacto-fish-vegetarian; SFAS, saturated fat and added sugars. Model I and Model II are adjusted for all the respective covariates in the table.
